# Soft, Transparent, Electronic Skin for Distributed and Multiple Pressure Sensing

**DOI:** 10.3390/s130506578

**Published:** 2013-05-17

**Authors:** Alessandro Levi, Matteo Piovanelli, Silvano Furlan, Barbara Mazzolai, Lucia Beccai

**Affiliations:** 1 Center for Micro-BioRobotics@SSSA, Istituto Italiano di Tecnologia, Viale Rinaldo Piaggio 34, Pontedera 56025, PI, Italy; E-Mails: alessandro.levi@iit.it (A.L.); matteo.piovanelli@iit.it (M.P.); barbara.mazzolai@iit.it (B.M.); 2 The BioRobotics Institute, Scuola Superiore Sant'Anna, Polo Sant'Anna Valdera, Viale Rinaldo Piaggio 34, Pontedera 56025, PI, Italy; 3 Department of Applied Mathematics and Theoretical Physics, Centre for Mathematical Sciences, University of Cambridge, Wilberforce Road, Cambridge CB3 0WA, UK; E-Mail: s.furlan@damtp.cam.ac.uk

**Keywords:** optical, artificial skin, electronic skin, tactile sensor, pressure sensor, pressure distribution, soft, flexible

## Abstract

In this paper we present a new optical, flexible pressure sensor that can be applied as smart skin to a robot or to consumer electronic devices. We describe a mechano-optical transduction principle that can allow the encoding of information related to an externally applied mechanical stimulus, e.g., contact, pressure and shape of contact. The physical embodiment that we present in this work is an electronic skin consisting of eight infrared emitters and eight photo-detectors coupled together and embedded in a planar PDMS waveguide of 5.5 cm diameter. When a contact occurs on the sensing area, the optical signals reaching the peripheral detectors experience a loss because of the Frustrated Total Internal Reflection and deformation of the material. The light signal is converted to electrical signal through an electronic system and a reconstruction algorithm running on a computer reconstructs the pressure map. Pilot experiments are performed to validate the tactile sensing principle by applying external pressures up to 160 kPa. Moreover, the capabilities of the electronic skin to detect contact pressure at multiple subsequent positions, as well as its function on curved surfaces, are validated. A weight sensitivity of 0.193 gr^−1^ was recorded, thus making the electronic skin suitable to detect pressures in the order of few grams.

## 1. Introduction

In recent years, touch screens have represented one of the major drivers for new technological developments in the field of flexible touch sensors suitable for extended surfaces [1,2]. Resistive and capacitive methods represent the leading approaches in the field. In this context, resistive sensors show advantages of low cost and low power consumption, but their drawbacks include a reduction of the light transmittance of the screen, as a result of the overlaying sensitive layer on the display, and the existence of a minimum pressure threshold required for touch detection. These sensors consist of a layer of conductive elastomer or foam and they suffer of a highly non-linear force-resistance characteristic that requires the use of signal processing algorithms and causes poor long term stability [3,4]. Capacitive sensing gained importance, especially in consumer electronics, but its drawbacks are the high costs and complexity of fabrication, power consumption, stray capacitance and lack of pressure detection, especially when this transduction method is applied to extended areas in consumer electronic devices. Moreover, the materials these sensors are made of cause a reduced light transmittance through the screen [1,2,5].

Looking more closely at actual touch panels, they detect only the position of the contacts and use this input for human-machine interactions. This kind of interface is suitable for most applications but others would benefit from a further degree of interaction. Possible 3D user interfaces include, for example: graphic applications, games, 3D virtual object manipulation tasks etc. Though 2D gestures could emulate the interaction with a third dimension, they can't provide a mapping as intuitive and direct as it is for the two dimensions on a surface, causing a complex and not natural interaction. Indeed, it has been reported that pressure based interactions improve usability. For example, pressure based keyboards can improve key click performance on touch screens [6]. Therefore, pressure detection can add the third touch dimension to user interfaces but some pressure sensitive technologies are not suitable for application to touch screens. As a matter of fact, in order to be integrated over the screen, touch sensors need to be not only pressure sensitive but also transparent. The features of the sensor (mechanical, optical) as well as its function (pressure detection) are both fundamental for the feasibility of a new technology in practical touch screen application.

Besides pressure sensing, it is desirable that touch sensors be flexible, thin and bendable so that they can be applied over curved surfaces or flexible displays. These features would enable new form factors for devices and innovative products: the electronic industry is working on the next generation of mobile devices that have pocket size but have wider screens that can be rolled. The dramatic push towards having flexible systems (including all related electronics) by the consumer electronics market is rapidly improving the available technologies for developing new tactile sensing systems in modern robotics, where additional stringent mechanical characteristics are required. In particular, the emulation of the mechanical characteristics of the biological skin model is one of the major goals for humanoid and rehabilitation robotics [7,8]. In parallel, it is noteworthy to highlighting that new classes of robots are being investigated [9,10] that will find uses in applications where conventional hard robots are unsuitable. They represent the emerging field of soft robotics, which will highly benefit from the development of soft and flexible smart skins, since these will endow the soft artifacts with the capability to interact with the environment. In the mentioned robotic research areas, in addition to flexibility, features like softness and stretchability represent the bottleneck towards real skin-like devices that can both be integrated in 3D systems and imitate nature when interfacing with the outside world, *i.e.*, have a suitable compliance at sensor/environment interface.

Among more consolidated transduction methods for touch sensors, like resistive and capacitive ones, optical approaches have been investigated and developed to the extent that new sensors have been commercialized [11]. Main important reasons in support of optical sensors include: they are immune to electromagnetic and electrostatic fields that are common in industrial environments; they are not affected by humidity; their signals can be easily multiplexed and integrated using light emitting sources and demultiplexed using photodetectors, making them a good candidate for potential large area electronics [12–18].

Optical sensors can be divided in two main categories: fiber Bragg based optical sensors and microbending optical sensors. The first ones are composed of optical fibers with internal Bragg grating that reflects narrow spectral components of the light emitted by a broad spectrum source. Strain/pressure and temperature can be detected by analyzing the Bragg wavelength shift of the reflected light. An example of these kinds of sensors is reported in Reference [12] where it presented a sensitivity of 2.1 × 10^−3^ MPa^−1^.

The second type of optical sensors consists of waveguides in which their microbending can alter the transmitted light. The determination of the light loss is used to detect the pressure. The use of flexible optical fibers allowed force discrimination below 1 N with a resolution of 0.1 N, and up to 30 N with 1 N resolution [13]. A resolution of 0.05 N for loads up to 15 N has also been registered with the same sensor approach [14]. Another optical waveguide sensor exploited a different configuration of crosslinked optical fibers embedded in a silicone elastomer allowing discrimination of pressure in the medium regime range 20–30 kPa [15]. A high sensitivity of 1 kPa^−1^ was obtained in the optical sensor with two plastic fibers separated by a compressible optical cavity made of Polydimethylsiloxane (PDMS) [16]. The deformation of the cavity with pressure changes the transmissivity of the device and therefore the pressure can be determined from the light intensity at the output. Like in the previously commented approaches, here as well there are some fabrication complexities, mostly due to the alignment requirements for the optical components. Therefore, these sensors are not easily integrated in an array, thus limiting their application over extended areas. An interesting method was to use a tapered optical fiber embedded into a PDMS-gold composite [17] that resulted in excellent pressure detection corresponding to a weight ∼5 grams. The limits of this work are the poor optical transparency, the complexity of fabrication and the fact that for covering a large area the taxels need to cover the entire surface and have to be read one by one.

In this work we present and apply a novel optically-based approach by using a soft, flexible and transparent PDMS waveguide having the two-fold function of mechanical substrate and waveguide material, with air as cladding. We address the design and fabrication of the full tactile sensing system embodying the applied principle. As a result the fabricated device is stretchable, bendable, and rugged, while the principle on which it is based is potentially extendable to large areas. The mechanical characteristics of the electronic skin are due to the properties of PDMS [19], which is a soft, conformable and compliant material that can conform to large areas and surfaces of complex and not planar shapes. It is a homogeneous and optically transparent material for wavelengths ranging from 235 nm to the near-infrared, and that makes optical detection possible over the entire visible region. Moreover, it has an attenuation as low as 0.4 dB/cm. While the air-PDMS-air configuration has been previously investigated [18,20] and flexibility and stretchability proposed [18], to the authors' knowledge the possibility to provide information about multiple contacts, pressure distribution and the related shape of occurred contact, by means of a complete smart skin was not addressed. Few artificial skins are more advanced since they can detect and reconstruct the shape of the contact, but they lack stretchability and are only partially flexible [21,22].

In the present investigation, we attempt to go beyond the state of the art by proposing a new concept of smart electronic skin (e-skin) that has: (1) intrinsic mechanical compliance, stretchability and flexibility; (2) the capability to provide information about multiple contacts and the distribution of pressure externally applied; and (3) the capability to retrieve information about the shape of the contact. Although this last point is not fully investigated in the present work, a preliminary validation of such aspect is provided.

The paper is organized as follows: in Section 2 we present the electronic skin concept, we illustrate the design and physics of the tactile sensing mechanism, its embodiment in a prototype, the electronics and the reconstruction method, as well as the experimental set-ups and protocols used in the characterization. In Section 3 the results of the preliminary experimental analysis to validate the concept are reported. Finally, in Section 4 and Section 5 the discussion and the conclusions are reported, respectively.

## 2. Materials and Methods

The electronic skin comprises a tactile sensing mechanism, conditioning and acquisition electronics, and reconstruction software running on a PC.

### 2.1. Tactile Sensing Mechanism: Design and Physics

The tactile sensing mechanism is based on a mechano-optical transduction principle. The intensity of an electromagnetic wave traveling in a waveguide is modulated by mechanical deformations of the waveguide itself. From the intensity measured at the boundaries of the waveguide, it is possible to reconstruct the location and entity of mechanical deformations, by solving an inverse problem by a process that is similar to tomographic backprojection [23].

The device in this work consists of a thin flexible elastomeric transparent layer embedding along its periphery electromagnetic emitters and detectors that are positioned in a known configuration. Because of the total internal reflection phenomenon [24], signals from the emitters are bound in the elastomeric layer and they reach the detectors. Given that n_1_ > n_2_, where n_1_ is the refractive index of the elastomeric layer and n_2_ is the refractive index of air (n_2_ = 1), the electromagnetic radiation emitted in the waveguide and incident on the boundary at an angle larger than or equal to a critical angle θ_c_, (Equation (1)) is completely reflected, and thus results bound in the guiding layer:

(1) $$\theta_{c}=\arcsin\left(\frac{n_{2}}{n_{1}}\right)$$

**Figure 1.**
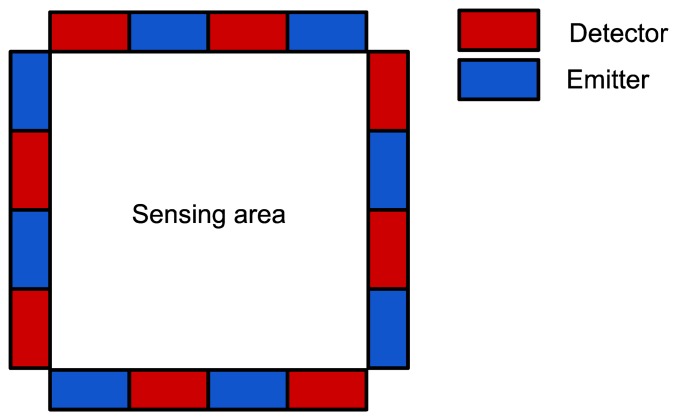
Schematic of the tactile sensing device layout.

Figure 1 provides a 2D schematic representation of the tactile sensing device showing the *sensing area* bounded by emitters and detectors with specific relative positions. In principle, the *sensing area* can have various shapes without any detriment to the operation of the sensor: however, this aspect is not addressed in the present investigation.

**Figure 2.**
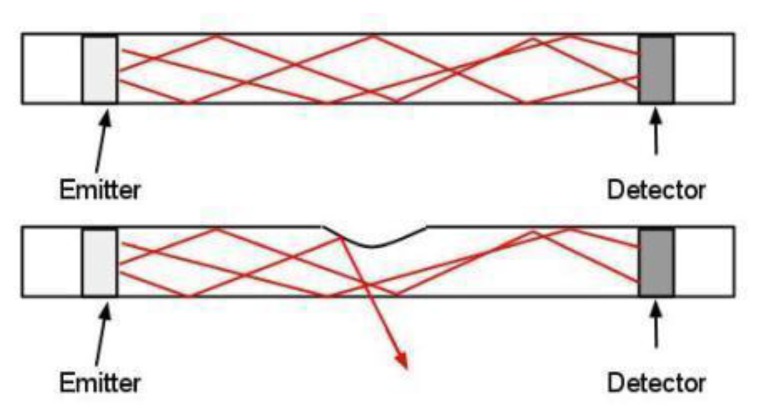
Schematic of sensor working principle in case an external mechanical stimulus: (**a**) is not applied; (**b**) is presented at the top of the sensor. Red lines show a travelling wave direction.

Figure 2 exemplify the operating mechano-optical transduction principle of the sensor. When no mechanical stimulus is externally applied, electromagnetic waves with a known intensity *J_0_* propagate to each detector from the emitters. The signal is then converted to a current *I_0_* which is then read and elaborated. When a mechanical contact event occurs, for example if an indentation is performed with an object on the surface of the *sensing area*, there is a variation in the output current *I* of the detectors, associated to both the contact area and the applied pressure of the applied mechanical contact.

The reason for this is that the mechanical contact causes concurrent effects that lead to a change in the intensity of the light reaching the detectors, since the electromagnetic waves are partially deflected out of the waveguide. This behavior happens because of two main mechanisms: (1) the *Frustrated Total Internal Reflection* effect [25–27] which is due to a variation of the refraction index caused by the contact; and, (2) the deformation of the compliant waveguide, which causes a loss of light intensity, similarly to the bending of optical fibers [28].

The quantitative determination of the pressure applied to all points of the waveguide surface is beyond the scope of this work. We assume, as a first approximation, that we are in a regime of small deformations, where the mechanical behavior of the waveguide can be considered linear. These deformations, whose amplitude then depends linearly on the applied pressure, cause changes in the curvature of the waveguide interface. The losses of electromagnetic intensity caused by the changes in curvature can be expected to be non-linear, similarly to what is observed when deforming optical fibers [28].

Starting from the variations of signal intensity recorded at the periphery of the waveguide, the determination of the deformations that cause the electromagnetic losses is an inverse problem. Conceptually, this problem is similar to those found in tomographic imaging. Therefore the reconstruction process we used is inspired by tomographic back-projection procedures [23]: however the formal treatments developed for those applications cannot transfer directly to our case, since geometry and conditions are too different. A back-projection process allows obtaining an image mapping the deformations on the whole surface: on this map it is possible to observe the position and intensity of the deformations.

A further aspect concerns the possibility to reconstruct the shape of the contact area. Although this aspect was not thoroughly investigated in this work, some preliminary results will be provided in Section 3.

In general, we can state that the relation between the light intensity collected by the detectors, *J*, and the pressure applied on the waveguide, *P*, is given by:

(2) $$P\left(i,j\right)=F\;\left(\sum_{1}^{M}\sum_{1}^{N}J_{K}\right)$$

where *F* is a generic function that can be used in a reconstruction algorithm in order to take into account the sum of light intensities emitted by all M light emitters and reaching each of the N detectors in the boundary. *P(i,j)* represents the pressure calculated in the specific pixel point *(i,j)* resulting from a discretization of the sensing surface that will be described in Section 2.4.

### 2.2. Sensor Fabrication

An electronic skin prototype was built embodying the tactile sensing mechanism described above. In such a system a polydimethylsiloxane (PDMS) waveguide structure is used with embedded emitters and detectors. The emitters are infrared (IR) LEDs, while the detectors are phototransistors (*i.e.*, photodetectors, PDs). The components were chosen based on the wavelength at which their performances peak match, in either case being 950 nm. PDMS was chosen since its optical and mechanical properties are well characterized [19]. However, we verified the refractive index of PDMS for different wavelengths performed by means of reflectometry. From the data, we could extrapolate a value of n_1_ = 1.428. Like explained in the previous section n_2_ is the refractive index of air, thus n_2_ = 1. Considering Equation (1) we obtain a critical angle θ_c_ = 44.45°. Therefore the LEDs chosen to build the electronic skin have a narrow cone of emission, mostly included between ±30°, hence a large part of their emitted power is transmitted in the planar waveguide.

**Figure 3.**
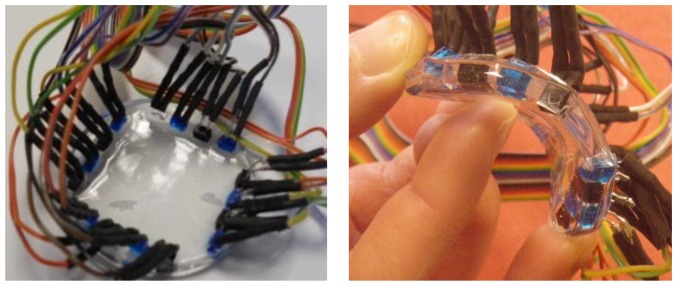
Electronic skin with external wiring: (**Left**) top view; (**Right**) side view when flexed.

The sensor was fabricated by embedding eight LEDs (TSKS5400S, 950 nm, Vishay, Malvern, PA, USA) and eight PDs (TEKT5400S, 950 nm, Vishay) in a 5 mm thick layer of PDMS having a diameter of 5.5 cm. The 16 components were fixed to a plastic support frame by their connectors, thus leaving the head of each component free. The components were then placed on their heads in a Petri dish. PDMS (Dow Corning Sylgard 184) was prepared by mixing the curing agent to the base monomer with 1:10 weight ratio and degassed in vacuum for about 1 hour. A suitable quantity of the liquid PDMS mixture was poured in the petri dish to reach the 5 mm thickness required to completely embed the active part of emitters and detectors in the polymer. Finally, the sample was put in an oven at 60 °C for 3 hours and the curing phase ended after approximately 12 hrs at room temperature. The resulting prototype is shown in Figure 3, where the blue components are LEDs and the black components are PDs.

### 2.3. Electronics

**Figure 4.**
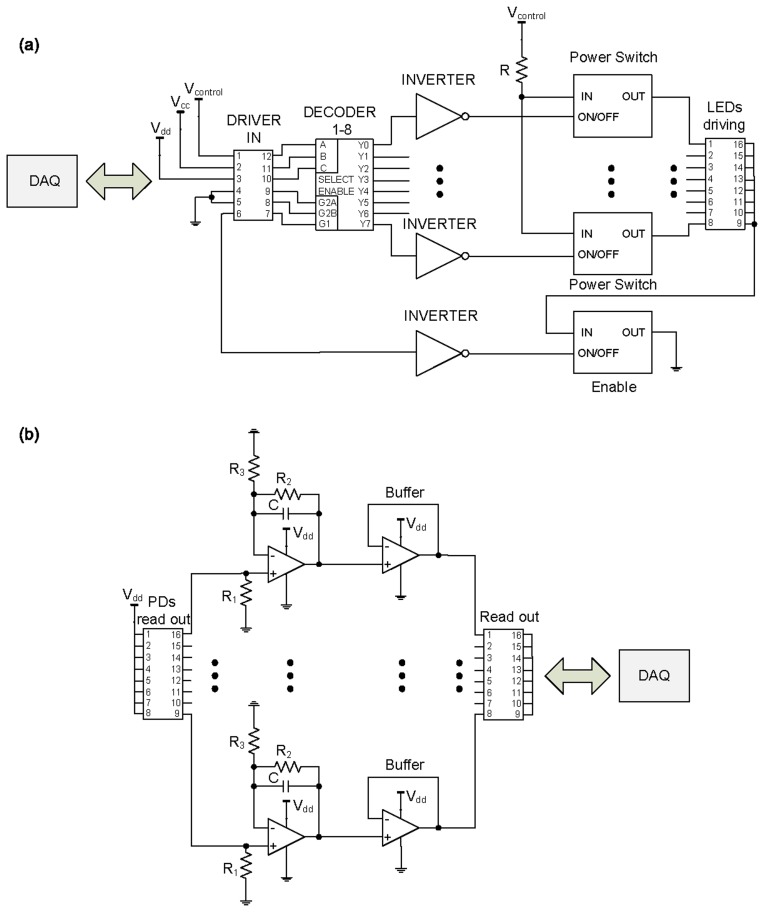
(**a**) Schematic overview of the driving electronics; (**b**) Schematic overview of the readout electronics.

The electronics (see Figure 4) designed for the proposed artificial skin consists of two independent parts on the same printed circuit board (PCB): driving electronics to switch on the LEDs in the desired way and readout electronics to acquire and condition the output signals from the PDs. Two power voltages were used: 3 V for both driving and readout electronics, and 5 V for V_control_, which was used to polarize the LEDs.

The driving electronics comprises a 1–8 decoder/demultiplexer (Texas Instruments CD74AC138M) whose outputs activate one power switch MOSFET (Fairchild Semiconductor FDC6330L) at a time. Since the decoder is active low, inverters (Fairchild Semiconductor 74AC04MTC) are used to drive the power switch MOSFETs. When the corresponding power switch MOSFET is activated, the LED is switched on and polarized with the required current, 10 mA to be working in the linear zone of its I-V characteristic. The polarization resistance R was therefore calculated to be 390 Ω. The schematic in Figure 4 shows how an additional power switch MOSFET (called Enable) is used to disconnect the common terminal of all LEDs from ground. This choice was taken to make the schematic of the electronics modular, so that it can be used with a greater number of active components. Using a specific configuration of four 1–8 decoder/demultiplexer, 32 LEDs can be sequentially activated one by one. This way it is possible to replicate the driving electronics a number of times, and sequentially activate a multiple of 32 LEDs.

The read-out electronics comprises a polarization stage for the phototransistors, a current-voltage converter stage that converts the photocurrents in voltages and finally an amplification and filter stage.

Considering a V_dd_ = 3 V, a collector-emitter saturation voltage V_CEsat_ = 0.3 V and a required collector current for the phototransistors of 4 mA, the polarization resistor R_1_ was set to 675 Ω. The gain of the amplifiers (R_2_/R_3_) was set to 1 by choosing for both R_2_ and R_3_ a value of 10 kΩ. The value for the gain should be chosen in order to avoid saturating the amplifiers' output with the highest electromagnetic signal coming from the LEDs. For future larger artificial skins the distance between LEDs and PDs would be higher, thus the gain will probably need to be set to a value larger than the unity. The refresh rate for the touch module was chosen to be 8 Hz and thus the clock frequency for the LEDs and PDs was 64 Hz. The low pass filter frequency was set to 38 Hz by choosing C = 415 nF, thus respecting the Nyquist theorem and avoiding back folded frequencies in the band of interest. After the amplifier/filter stage the outputs pass through a stage with op-amps in buffer mode and finally are sampled by the Data Acquisition (DAQ) system.

### 2.4. Reconstruction Process

The reconstruction process is inspired by tomography and consisted in the definition of three types of matrices and in addressing a backprojection procedure. The major steps in the reconstruction are described in the following.

The data acquired from the DAQ board is initially stored as a matrix, *A*, of double precision values. Each element in the line is the value acquired from a PD, with each line representing a period of activation for one specific LED. In the specific case of the experiments presented here and performed with the sensor described above, the resulting matrix has eight columns, one for each PD, and 8 N lines, where N is the number of times each LED has been activated, corresponding to the number of times the entire *sensing area* (see Figure 1) has been scanned. A similar matrix, *B*, obtained from acquisitions performed on the sensor during which there have been no contacts, is used to determine calibration values for all LED-PD pairs.

These two matrixes, *A* and *B*, are fed to a C++ algorithm alongside geometrical information about the relative position of all active components in the sensor. The geometrical information is used to generate an internal matrix representation, *C*, of the *sensing area* and compute correlations between each LED-PD pair and the points of that matrix. The correlations are used in the reconstruction process to determine whether the acquired values for any given LED-PD pair are related to contacts at a specific point or not. In the present work we used the simplest correlation approach: two parallel lines connect the extremities of the LED and the PD in each LED-PD pair, defining a trapezoidal section of the *sensing area*. For the reconstruction, only the points inside the trapezius are considered affected by the specific LED-PD pair.

The calibration matrix *B* is used to normalize the acquired matrix *A*, so that data is in the range between 0 and 1. This is followed by the backprojection process that consists in reading each value for each line from the normalized matrix *A/B*, corresponding to the measurement for a specific LED-PD pair, and adding it to the points of the matrix representation *C* determined in the correlation step. After having backprojected as many lines as there are LEDs in the sensor (eight in the case of the sensors used for this work), a contact intensity map of the entire surface at a given time has been computed and is saved. The images of the reconstructed *sensing area* in this paper are obtained by plotting the reconstructed maps using MatLab.

### 2.5. Experimental Tests

In a first phase, experimental trials were performed to test the electronic skin's working principle in order to provide significant information for the implementation and optimization of the reconstruction algorithm. This included loading tests performed on the sensor during which its output signals were analyzed without using the processing algorithm.

In a second phase, the aim of the experimental analysis was to test the capability of the electronic skin system (comprising the tactile sensing mechanism, its electronic conditioning system and the processing algorithm) to detect tactile information related to a mechanical stimulus. In particular, the position and intensity of contact were obtained. Moreover, preliminary trials for detection of one type of contact shape were addressed.

In a third phase, we performed preliminary experiments to begin validating the more advanced features of the electronic skin: detection of multi-pressure contacts and operation on curved surfaces. The experimental apparatus and protocols used in these tests are described in the next sections.

#### 2.5.1. Experimental Setup

**Figure 5.**
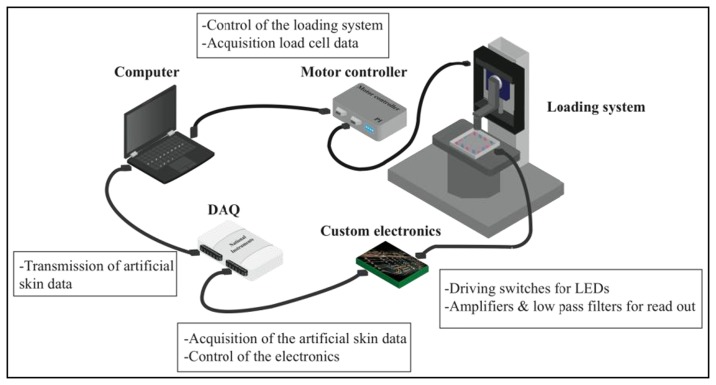
Block illustration of the experimental set-up for the artificial skin system. The loading system is depicted in detail in Figure 6.

**Figure 6.**
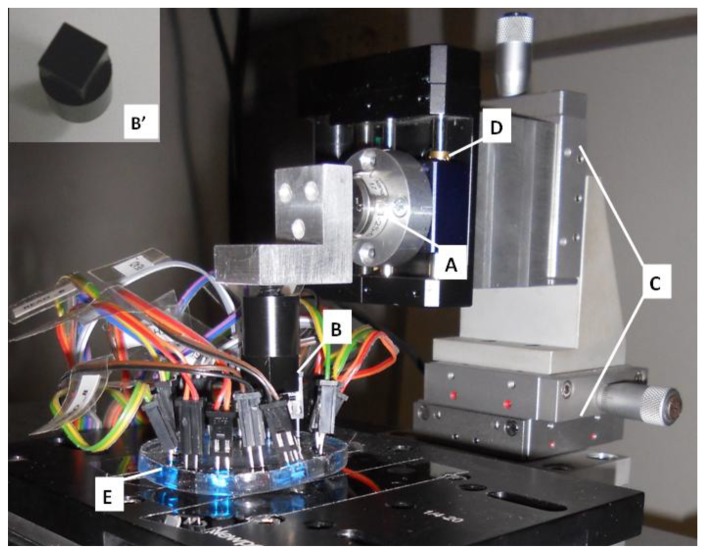
Image of the experimental set-up loading system, integrating: (**A**) the 3- axis load cell; (**B**) the Delrin loading probe whose 10 mm × 10 mm square shape is shown in (**B’**); (**C**) the three orthogonal manual micrometric translation stages; (**D**) the servo controlled micrometric translation stage. The 5 mm thick transparent electronic skin is shown (**E**), whose components are wired to the custom electronic system as schematized in Figure 5.

The experimental apparatus employed for the characterization of the smart skin system consisted of the components schematically illustrated in Figure 5. In particular, the loading system is shown more in detail in Figure 6. The force applied to the sensor was measured and recorded through a 6-axis load cell (ATI NANO 17F/T, ATI Industrial Automation, Apex, NC, USA) (A) interfaced to a loading probe (B). The vertical position of the load cell was determined with an initial rough manual positioning, by means of three orthogonal manual micrometric translation stages with crossed roller bearing (M-105.10,PI, Karlsruhe, Germany) (C), followed by an accurate controlled positioning, by means of a servo-controlled micrometric translation stage (M-111.1, PI) (D). This way the contact of the loading probe on the *sensing area* (see Figure 1) of the sensor (E) was achieved. In this work a probe with a square head (10 × 10 mm^2^) was chosen because the limited resolution of the artificial skin imposed a constraint to the reconstruction of polygons with higher numbers of facets. Indentation experiments with probes having different head shapes will be addressed in a future work with a higher resolution skin.

The electronic and acquisition system, integrated in the experimental set-up, consisted of (see Figure 5): a NI-DAQ board (USB 6216), that was used for generating the driving signals for the LEDs and for acquiring the electronic signals from the PDs of the sensor; an ad-hoc printed circuit board (PCB) designed for amplification and filtering of such signals, and for driving the LEDs; finally, a laptop with a C++ algorithm that performs the elaboration and outputs the contact/pressure distribution. When addressing the initial testing phase of the sole optical tactile sensor without the processing algorithm, the same loading system described above (Figure 5) was used, but not the acquisition section (filtering and amplification) of the electronics: rather the sensor outputs were acquired by an oscilloscope (Agilent Technologies MSO7014A) directly connected to the sensor and analyzed using Matlab software.

For the final test on the multi pressure reconstruction capability the set-up of Figure 5 was used but the loading system was substituted by an electronic scale where the optical sensor was set for the measurements.

In order to address a preliminary validation of the performance of the optical sensor on curved surfaces (as it will be explained in Section 2.5.2 (5)) a half cylinder tube was positioned on an electronic scale and loaded manually.

#### 2.5.2. Experimental Protocols

Different tests were performed to assess the overall performance of the sensor, according to the following protocols.

##### (1) Preliminary Experimental Analysis on the Sensor Working Principle

Acquisitions were performed without any load on the *sensing area*, with the aim to retrieve the shape of the analog signal that each PD produces when incoming electromagnetic signals, from each LED, reach them. The study of this analog signal is important because the core of the reconstruction algorithm has been developed from the analysis of variations of its shape. In particular, each column of matrix A (as defined in Section 2.4) contains a digitalization of this signal for a PD.

The output of one arbitrarily chosen PD was acquired directly with an oscilloscope during one driving cycle in which all LEDs were activated one at a time. No load was applied in this case and thereafter the measured signal could be considered as the offset state for the tested PD. The refresh rate of the sensor response was 8 Hz and thus each LED was turned on for 125 ms at a time.

##### (2) Indentation Tests

Loading tests were performed to assess the variation of the outputs of the PDs in function of externally applied loads and contact positions. In particular, forty different loads were applied, in the range from 0 kPa to 160 kPa. The probe chosen had a square shape of 10 × 10 mm^2^. The two contact positions tested and the two PDs considered are shown in Figure 9 and Figure 13. For each loading condition 10 outputs for each PD were acquired by the oscilloscope and the average of the waveform values was calculated with Matlab.

##### (3) Pressure Map Reconstruction

The artificial skin functionality was validated by means of indentation tests with a Delrin probe having a square head (area 10 × 10 mm^2^) as indenter, and by applying different values of normal load from 0 N to 80 KPa. The experimental set-up used is described in Section 2.5.1 and depicted in Figures 5 and 6. Four subsequent contacts, in four different positions of the *sensing area* (see Figure 16(a)), were executed for each acquisition sequence.

##### (4) Preliminary Multi-Pressure Tests

In order to give clues on the capability of the artificial skin to detect multiple contacts at the same time, the system was placed on an electronic scale, and four contacts were achieved on its *sensing area* by means of hand-held probes with a square section. Each contact was added sequentially, until four of them were on the surface, with the additional value measured by the scale after each new contact converted into a nominal pressure by dividing it by the known section of the probes. A multi-probe indentation setup is required to obtain a thorough characterization, but in a first validation phase a preliminary experiment was performed.

##### (5) Preliminary Pressure Map Reconstruction on Curved Surfaces

The response of the artificial skin positioned on a curved surface of 5 cm bending radius was tested. In this test a half cylinder structure was positioned on an electronic scale and the load was applied with a Delrin square shape probe (area 1 cm^2^). A loading pressure of about 142 kPa was applied by making four subsequent contacts, each time releasing the load and waiting for 5 seconds between contacts. As in the previous case (*i.e.*, (4)) this represents a preliminary validation.

## 3. Results

The results of the experiments which protocols are described in the previous section are reported in the following in separate sub-sections.

### 3.1. Preliminary Experimental Analysis on the Sensor's Working Principle

**Figure 7.**
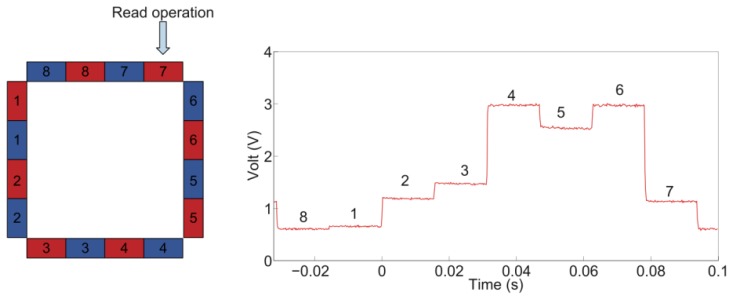
Photovoltage measured at PD 7 when no load is applied. The numbers in the plot on the right indicate the LED responsible for the corresponding part of detected signal.

The photovoltage output of the detectors was measured when neither physical contact nor load was applied on the *sensing area* of the artificial skin system. Therefore, Figure 7 shows the voltage acquired by the oscilloscope across the marked PD, *i.e.*, PD 7. The recorded photovoltage spans from 0.6 V to 3 V. This curve is an example of the calibration voltage (or offset) used in the process of reconstruction of contacts on the *sensing area*. Moreover, this kind of output represented the reference voltage, V_0_, used to calculate the voltage variation when a load was applied on the *sensing area* surface with an indenter.

**Figure 8.**
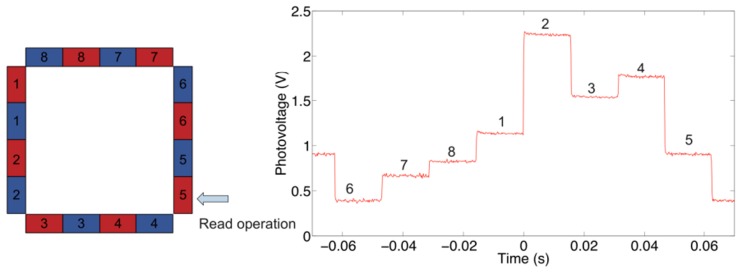
Photovoltage measured at PD 5 when no load is applied. The numbers in the plot on the right indicate the LED responsible for the corresponding part of detected signal.

Also, Figure 8 shows the photovoltage across PD 5 in absence of any contact or load applied. Owing to the different position of this PD with respect to PD 7, this photovoltage waveform is different from the one in Figure 7 and the voltage spans between 0.4 V and 2.25 V.

### 3.2. Indentation Tests

Here we show the results of the indentation experiments by considering the waveforms of the photovoltage across the same detectors of the measurements described in the previous section, *i.e.*, PD 5 and PD 7. In particular, we focus on the results obtained when the signal from a specific active emitter showed the largest variations at the detector. Moreover, the emitter-detector pair for which the results are shown in the following, highlight two typical cases of contact occurring on the *sensing area*: in one case, contact occurs in the trapezius that corresponds to the portion of waveguide directly linking the emitter and detector; in a second case, contact occurs in a region only partially in the trapezius connecting the two components.

**Figure 9.**
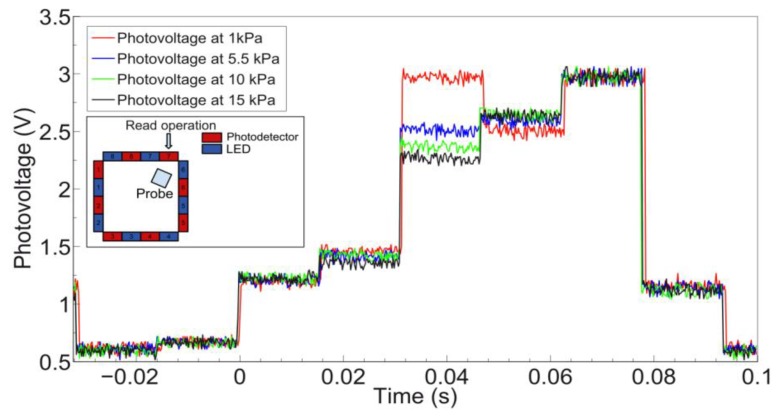
Waveforms of photovoltage measured at PD 7 when different loads are applied. The contact area is 100 mm^2^. Inset: schematic of the experiment.

Figure 9 shows some of the typical waveforms acquired across PD 7. Waveforms were acquired for 40 values of pressure, nevertheless in the graph four of them are reported for the sake of readability. The inset in Figure 9 contains a schematic representation of the conditions of the experiment, highlighting the relative position of indenter and detector.

**Figure 10.**
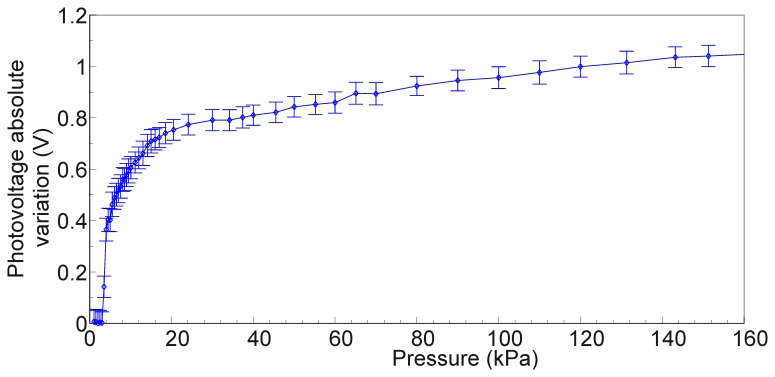
Absolute variation of photovoltage, (V_meas_ – V_0_), at PD 7 receiving the signal from LED 4, with error bar.

The largest voltage variation at PD 7 was observed when the active LED was number 4. Therefore this is the case in which the contact occurs on the portion of the *sensing area* directly linking the emitter (LED 4) and detector (PD 7). The absolute voltage variation (V_meas_ – V_0_) versus the applied load for the pair defined by LED 4 and PD 7 is shown in Figure 10. As previously explained (see Section 3.1), V_0_ is the voltage measured at the output of PDs when no contact or external load is applied.

**Figure 11.**
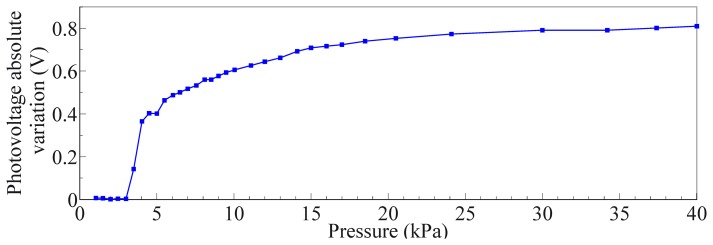
Absolute variation of photovoltage, (V_meas_ – V_0_), at PD 7 receiving a signal from LED 4.

Figure 11 shows a magnification of the same curve in Figure 10, focusing on the 0–40 kPa pressure range. It can be noticed that at 40 kPa the absolute photovoltage variation is 0.8 V. We can define the sensitivity as the slope of the photovoltage relative variation, ΔV/V_0_, versus the the applied pressure, P, as follows:

(3) $$S=\frac{1}{\frac{\Delta \;V}{V_{0}}}\frac{d\;\left(\frac{\Delta \;V}{V_{0}}\right)}{dP}$$

**Figure 12.**
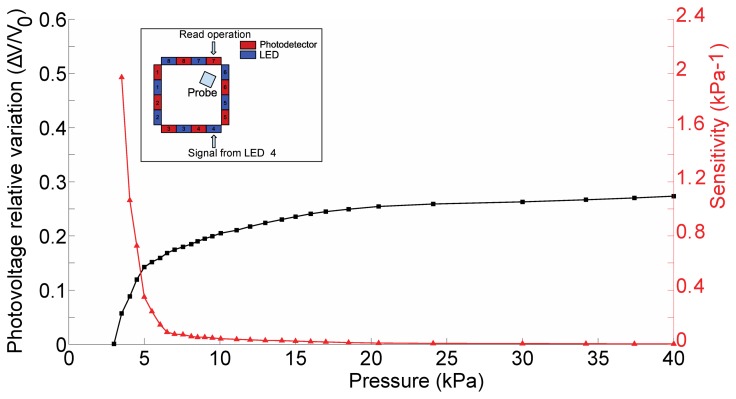
Relative variation of detected photovoltage, (V_meas_ – V_0_)/V_0_, and sensitivity for the pressure optical sensor using PDMS as bulk material. The read-out operation considers PD 7 receiving light from LED 4.

Accordingly, Figure 12 shows the sensitivity as well as the relative photovoltage variation for the pair represented by LED 4 and PD 7.

**Figure 13.**
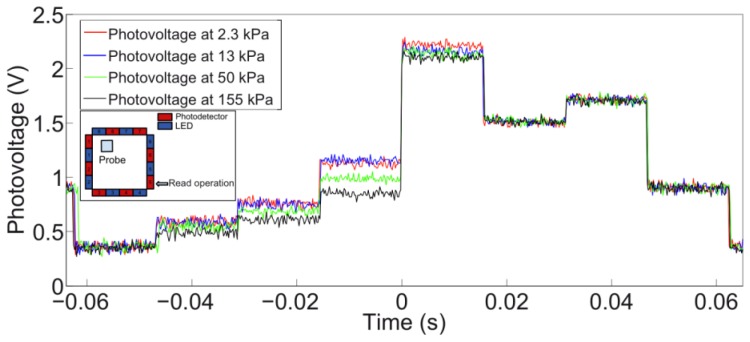
Photovoltage measured at PD 5 when different loads are applied. The contact area is 100 mm^2^. Inset: schematic of the experiment.

In considering the results obtained for PD 5, Figure 13 shows some of the waveforms acquired across such detector. As in the previous case, while 40 values of pressure were used, the output signals resulting from four indentations are reported in the graph for the sake of readability. The inset in Figure 13 contains a schematic representation of the conditions of the experiment, with the approximate relative position of indenter and the contact area with respect to PD 5.

**Figure 14.**
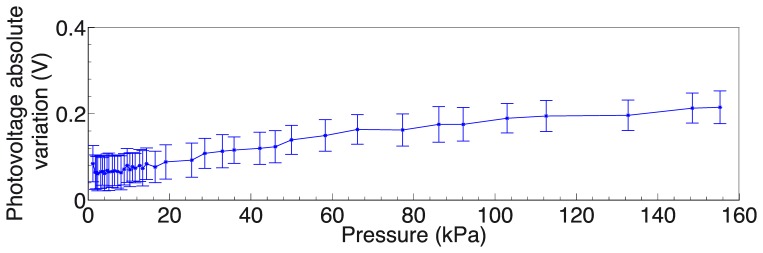
Absolute variation of photovoltage, (V_meas_ – V_0_), at PD 5 receiving signal from LED 8, with error bar.

The largest photovoltage variation was observed when the active LED was number 8. Therefore, in this case contact occurs in a region only partially in the trapezius connecting emitter and detector. The absolute voltage variation (V_meas_ – V_0_) versus the applied load for the pair defined by LED 8 and PD 5 is shown in Figure 14.

**Figure 15.**
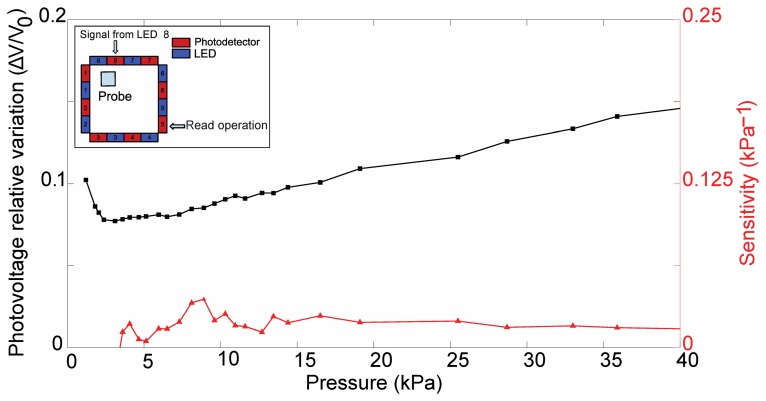
Relative variation of detected photovoltage, (V_meas_ – V_0_)/V_0_, and sensitivity for the pressure optical sensor using PDMS as bulk material. The read-out operation considers PD 5 receiving light from LED 8.

Figure 15 shows the sensitivity, defined as the slope of the photovoltage versus the applied load,in Equation (3) as well as the relative photovoltage variation, for the pair represented by LED 8 and PD 5.

### 3.3. Pressure Map Reconstruction

**Figure 16.**
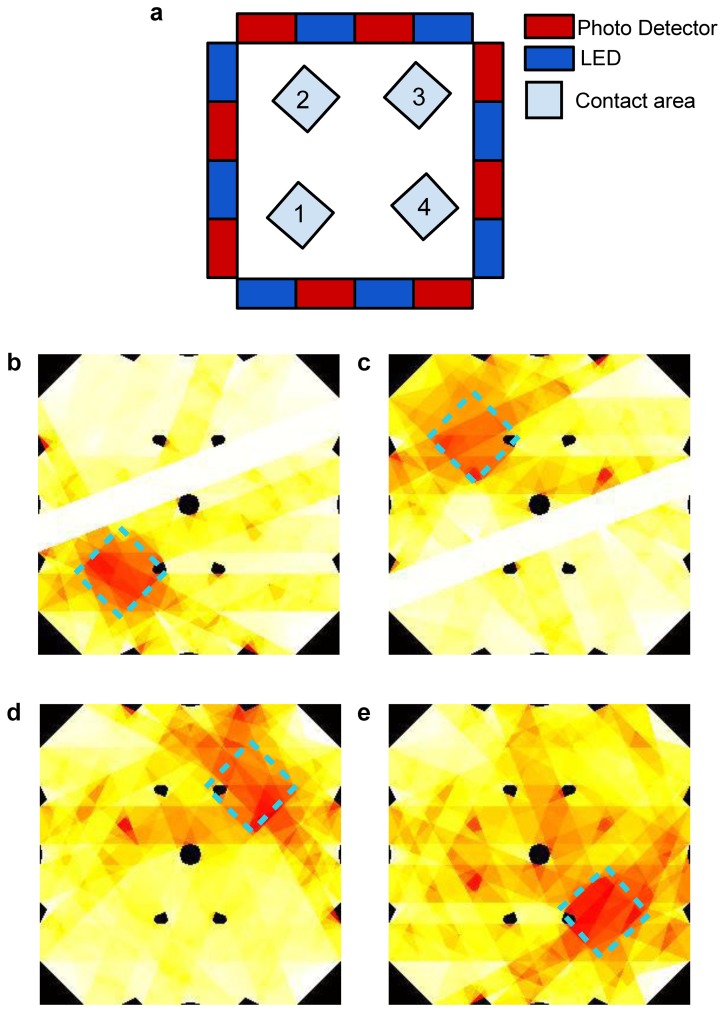
(**a**) Design layout of the sensor with the approximate position of four subsequent contacts depicted; (**b**–**e**) the corresponding two dimensional intensity profiles obtained from reconstruction, in which the square shape corresponding to the applied pressure profiles is highlighted, and the probe contact positions can be noticed.

This experiment focused on the performance of the electronic skin in mapping pressure applied to its *sensing area*. Figure 16 shows selected frames obtained through the reconstruction algorithm when the applied pressure was 70 kPa. The white stripes and black spots in the frames shown in Figure 16(b–e) are artifacts introduced by the reconstruction algorithm because of the LED-PD geometrical correlation approach chosen. To overcome these issues a coupling scheme more sophisticated than the trapezoidal approach should be used, allowing interpolation of the information on the sensing area. Alternatively, these artifacts could be reduced by using more components, possibly smaller than those used in this work, and positioned closer to each other. It is worth pointing out that no filtering of the signals was performed during or after the reconstruction. Thus, the image shows the raw data obtained through simple reconstruction, without elaborating the signals to improve the quality of the results.

### 3.4. Preliminary Multi-Pressure Tests

**Figure 17.**
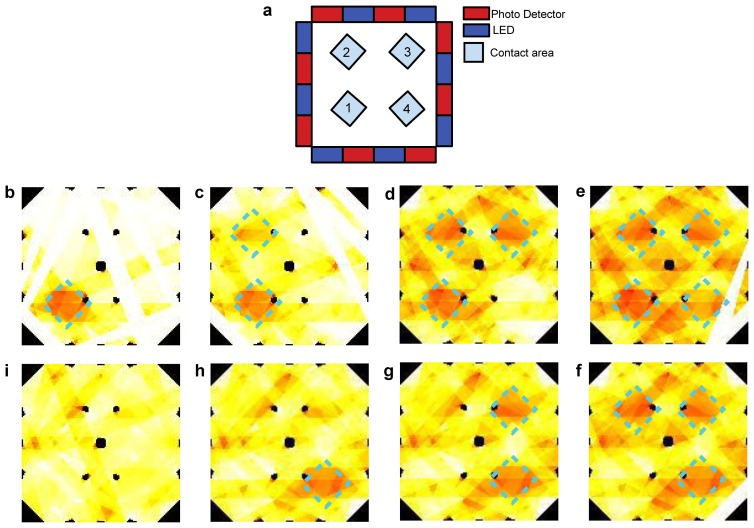
(**a**) Design layout of the sensor with the position of 4 subsequent and multiple contacts depicted. At bottom and clockwise: (**b**–**e**) show the corresponding two dimensional intensity profile obtained through the reconstruction by mapping the pixels signals, in which the multiple contacts square shape, corresponding to the applied pressure profile, can be reconstructed by the e-skin, as well their different positions; (**f**–**i**) show the subsequent release of the square shape contacts as detected by the e-skin.

This kind of experiment aimed at giving preliminary results for the multi-pressure contact detection of the electronic skin. Figure 17 shows selected frames obtained through the reconstruction algorithm. As explained in Section 3.3, the white stripes and black spots in the frames shown are artifacts introduced by the reconstruction algorithm, and here as well no data filtering was performed during or after the reconstruction.

### 3.5. Preliminary Pressure Map Reconstruction on Curved Surfaces

**Figure 18.**
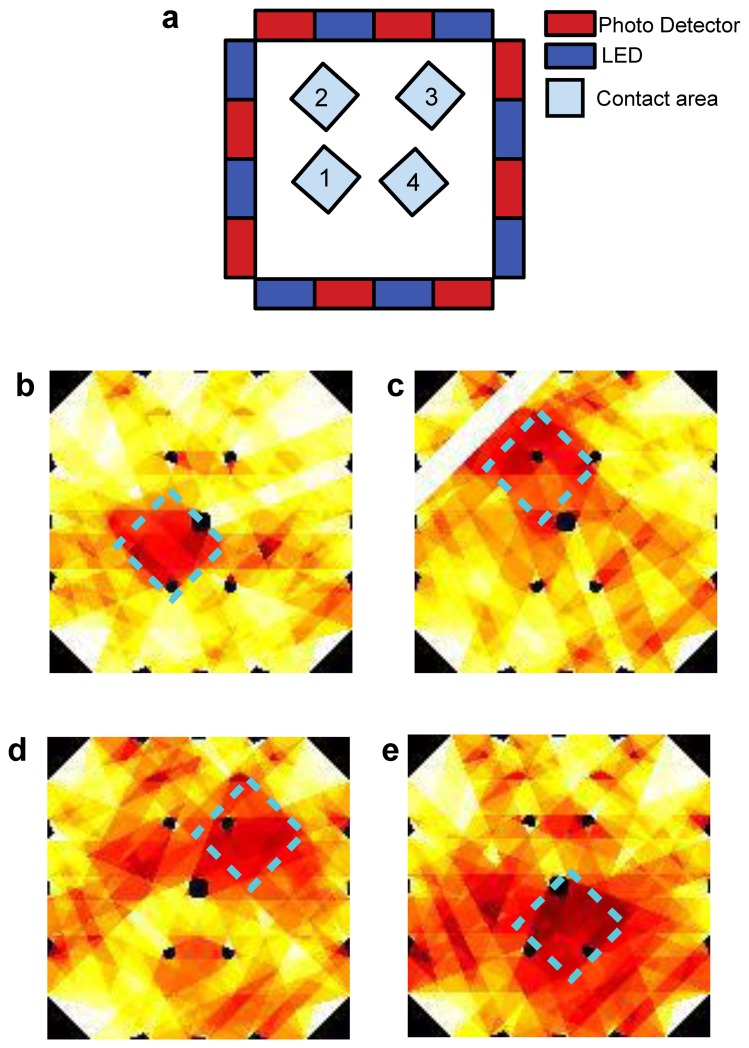
(**a**) Design layout of the sensor with the position of four subsequent contacts depicted; (**b**–**e)** The corresponding two dimensional intensity profile obtained through the algorithm by mapping the pixels signals, in which the square shape corresponding to the applied pressure profile can be reconstructed by the e-skin positioned on a curved surface, as well its different position.

This experiment was performed with the goal of showing that the electronic skin can operate even when bent on curved surfaces. Figure 18 shows selected frames obtained through the reconstruction algorithm. The same artifacts described in Section 3.3 are evident. Also in this case, no filtering was performed during or after the reconstruction.

## 4. Discussion

The design of the system investigated in this work aimed at an electronic skin of simple fabrication overcoming some limitations of current planar pressure sensor technologies. This was achieved by the adoption of a mechano-optical principle of detection, and the use of a distributed peripheral architecture for the active components. The fabrication process is simple and repeatable as it uses PDMS as bulk material for the waveguide and through-hole components as active elements. Differently by other optical sensors approaches [17] this sensor is based only on a PDMS film functioning both as waveguide and substrate. Two concurrent phenomena are responsible for the working principle of the sensor: (1) the deformation of the flexible waveguide upon application of a pressure on the sensing area; and (2) the out-coupling of wave-guided signals at the position of the deformation. To our knowledge, only in Ramuz *et al*. [18] we can find an approach exploiting the same physical principles, hence that work will often be referred to in the present discussion. It is worth pointing out that the authors of that work considered a single emitter and a single detector, and as a consequence its results are only relevant to the first part of the present work, and not where we used the whole distributed system to reconstruct maps of the sensing area.

An immediate consequence of the used transduction method and related electronic skin architecture is the fact that no active component is in or under the sensing area. Furthermore, the latter is a planar waveguide transmitting the signals from the emitters to the detectors, and so there is no need for connections or wiring to cross it. Both these design choices allow high transparency and high flexibility of the sensing area, and are at odds with what is generally seen in planar pressure and touch sensor technologies, in which active components are sensing “pixels” in the sensing area, and thus need connections to cross it. Moreover, an important aspect related to the architecture of the tactile system is that the number of active components increases linearly with the length of the sensing area periphery, rather than with its area. This can prove to be an advantage in terms of both costs and power consumption. On the other hand, this architecture places a larger burden on the system in terms of computations required to correctly evaluate contacts on the sensing area, because this information needs to be determined indirectly.

It must be noted that in the design of the system, the thickness of the electronic skin plays a key role, since it needs to strike a balance between flexibility, sensitivity and robustness. In the present work, such parameter was mostly affected by the choice of simplifying the optical coupling between components and the waveguide by entirely embedding the former in the latter. Thus the PDMS waveguide was 5 mm thick.

Concerning the specific results that were obtained from the indentation experiments, in the following we discuss the two typical cases considered in Section 3.2. The largest variations of photovoltage with respect to increasing load were observed for emitter-detector pairs, respectively 4–7 and 8–5, for which the deformation affected the most the path of transmission of the IR signal in the planar waveguide. Conversely, we can see almost no variation in the signal from LED 6 to PD 7 when the probe is in the position specified in Figure 9, as would also be expected tracing a ray connecting the two components and observing that it does not intersect the region affected by the indentation. In the data on the LED 4 - PD 7 pair (see Figures 9, 10, 11 and 12), we see a lack of response for the initial points for which the load applied was low. This behavior probably originates from the fabrication of the electronic skin, and the chosen position of the indentation probe. Indeed, the thickness of the PDMS waveguide (*i.e.*, 5 mm) was chosen in order to completely embed the “heads” of the electronic components in the material. However, the sensitive portion of the phototransistors is actually a little smaller than the whole immersed portion, leading to the fact that a portion of the signal reaching the component, rather than being detected, is lost where it “hits” the packaging of the component. Bearing that in mind, when the indentation experiment is performed by positioning the probe just in front of the component (as in the case we are now referring to) increasing the load does indeed reduce the optical transmission channel, but it does not affect the signal portion that reaches the actual sensitive portion of the component. After the first few points in which the lack of response is apparent (see Figures 9, 10, 11 and 12), further increases in load cause a reduction in the photovoltage out of the phototransistor, PD 7, as was expected. The just described behavior is not the same when the indenting probe is positioned far from the detector, as is for example the case with the other LED-PD pair reported in Figures 12, 13 and 14.

Besides the above considerations, the trend observed in Figures 9, 10, 11 and 12 for the relative photovoltage variation for the LED 4–PD 7 pair is similar to the one reported in Reference [18]. An important difference is in the amount of variation of the signal for a specific pressure range: while Ramuz *et al*. report a variation of almost 90% around 35 kPa, in our experiments we observed a 25% variation for a similar load. The most probable reason for this behavior is to be found in the thickness difference between the two systems: while the present electronic skin is 5 mm thick, in Reference [18] a waveguide of 600 μm thickness is employed. As a result, the electronic skin presented in this work suited also for higher pressure regimes: indeed, photovoltage variations could be appreciated up to the application of 160 kPa. At a first rough approximation this can be explained by invoking the fact that a thicker waveguide can support a larger number of guided optical modes. However, in increasing the pressure range a lower Signal-to-Noise Ratio (SNR) is obtained that translates in a lower sensitivity (as from Equation (3)): we see a peak of 1.93 kPa^−1^, while in the reference they showed sensitivity up to 0.2 kPa^−1^. These sensitivities are calculated using the pressure, and are thus dependent on the surface in contact with the probe. A possibly better parameter for comparison is the weight sensitivity, independent from the contacting surface, expressed in gr^−1^. Our measured peak sensitivity of 1.93 kPa^−1^ corresponds then to a weight sensitivity of 0.193 gr^−1^ while in Reference [18] a higher weight sensitivity of 30 mg^−1^was reported. This makes the electronic skin suitable to detect a measured weight in the gram range.

Furthermore, concerning the relation obtained in the photovoltage-pressure graph of Figure 12, it can be noted that it is typical of an IR light beam (bundle) crossing the contact area and consists of a power law. Such relation derives mainly from geometrical considerations of the deformation of the waveguide surface responsible of the total internal reflection phenomenon, an issue currently under investigation. Additional effects, like the proximity of the contact area to the photodetector, can affect the relation especially in the lower pressure regimes. When the light beam bundle partially crosses the contact area the sensitivity is proportionally reduced due to a reduced number of light beams sensing the deformation. The reduction of sensitivity comes with the scaling of the photovoltage-pressure graph shown in Figure 15: basically, we can argue that the curve in Figure 15 could be the first portion of a curve exhibiting the same power law shown in Figure 12, but scaled with respect to pressure. However, more experiments are required to be sure on this point.

Still regarding the LED 8-PD 5 pair, in Figure 15 we see that the sensitivity, albeit lower as was just discussed, is more or less constant on the whole pressure range. This is symptomatic of an almost linear response, most likely related to the closeness of the emitter to the contact position. There is a correlation between the spatial position of the pressure location and the sensitivity. This happens because the PDMS works as a planar waveguide and light travels in all directions. When the pressure location is far away the light source, the percentage of light intercepted is lower and this means to have a lower dampening percentage. If the pressure location is near the light source, more light can be dampened by the applied pressure and there will be a higher dampening percentage.

Concerning the experimental analysis performed to assess the capability of the system to provide a pressure map, we refer in the following to the results shown in Figures 16, 17 and 18. Because of the low number of emitters and detectors used to build the electronic skin tested in this work, we did not expect a good spatial resolution for the contacts on the sensing area. The reconstruction we used to determine contact information is similar to backprojection reconstructions used in tomography, for which there is a large volume of theory showing that effective resolution, represented by number of significant reconstructed pixels, is limited to the square of the number of emitters (or detectors) [23]. While it would not be correct to directly transfer those theories on our system, because of the different geometries involved, the artifacts we find in our results are not unlike those resulting from low number of emitters (or detectors) in classical tomography. Indeed, in all the images obtained from our reconstructions (see Figures 16, 17 and 18) it is easy to see stripes and black spots introduced by the particular algorithm used. A further cause of the artifacts in the reconstructed results is the particular coupling scheme chosen for emitter-detector pairs. While the use of trapezii to define the points of the sensing area affecting the signal detected for a specific pair is the simplest to implement algorithmically, its lack of any smoothing or interpolation introduces the sharp edges of the stripes in the figures. The use of a larger number of components, along with the introduction of more advanced coupling schemes for emitter-detector pairs can in the future improve on the spatial performances of the electronic skin presented in this work.

Further limits to the spatial resolution of the electronic skin are posed by the material used to fabricate the planar waveguide. The deformation of the surface cannot be expected to exactly follow the contact profile; rather, a mechanical filtering effect is introduced that tends to smear the boundaries of the detected contact. This effect can be somewhat mitigated, if the material used is well characterized, by a post-processing step in the reconstruction algorithm. However, in this work the limits to the resolution introduced by the low number of components and by the coupling scheme are such that the mechanical effect could not be appreciated.

Bearing in mind all these considerations, the results in Figures 16, 17 and 18 still show quite accurately the position of the contacts applied on the sensing area. Moreover, the shape of the probes used can clearly be seen. A discussion similar to the one made for the limits of the system in this work in terms of resolution can also be made regarding the ability of the electronic skin to determine the shapes of applied contacts. In the pressure map experiments in this work we limited ourselves to square probes. Nonetheless, the obtained results are remarkable when compared to a different state-of-the-art e-skin [21] that was able to reconstruct the contact profile of a larger probe (3 cm^2^) using a 19 × 18 sensor array: the electronic skin in this work only uses 16 components (eight emitters and eight detectors). This comparison is useful to underline the strength of the approach and architecture chosen for the electronic skin compared to planar sensor arrays [21,29–31].

Even if the results were obtained through a preliminary analysis, Figure 18 shows that the electronic skin allows the detection of multiple simultaneous contacts on its sensing area. It can be construed that using a higher number of emitters and detectors, the present electronic skin principle should ideally allow the detection of any number of contacts. Limits to that can be introduced by the materials used, because the mechanical filtering effect introduced above might prevent discrimination of contacts that are too close one to the other, by signal noise and by system constraints, like for example the maximum allowable data rate in a real time system. Moreover, a theoretical limit to performance is also present in the tomographic theories mentioned above [23], where SNR is defined proportional to $\sqrt{E}/D^{3/2}$, with E being the number of emitters and D the number of detectors.

The importance of fabricating a flexible and compliant electronic skin is strongly related to the possibility of detecting tactile cues when the device is on a curved body. This opens a plethora of applications in robotics and for consumer goods that can present this way new form factors. The preliminary experimental analysis performed so far by positioning the device on curved surfaces can thus provide an initial validation of the system, and results are shown in Figure 18. This is allowed by the fact that any condition of stress and deformation of the sensing area can be used to determine a calibration state for the system. This is simply realized by using the photovoltage values for each emitter-detector pair acquired when the sensing area is in the desired condition in the calibration matrix used by the reconstruction algorithm. As a consequence, the electronic skin does not need to be used on a flat surface.

## 5. Conclusions

The integrated electronic skin proposed in this work is rugged, but still has high mechanical flexibility and it is easy to fabricate. The mechano-optical sensor showed high transparency since we used a PDMS layer as both a waveguide embedding the active components and as the sensing area of the e-skin.

The artificial skin was tested by applying pressures up to 160 kPa. In such a range output voltage variations could be appreciated and this demonstrates the suitability of the e-skin for touch interfaces. In fact, normal forces in the range of 0 N to 2 N are most relevant when a fingertip makes a dynamic contact with a flat surface during a typical exploration task [32] similar to that performed when interacting with a touch screen. Therefore, if we were to consider a contact area of the fingertip of 1 cm^2^, the pressure involved would range from 0 to 20 kPa, while higher pressures (e.g., up to 100 kPa) would be used in case the contact area is smaller (e.g., down to 0.2 cm^2^).

We found a peak sensitivity of 1.93 kPa^−1^ which corresponds to a weight sensitivity of 0.193 gr^−1^ for an emitter- detector pair, which makes the electronic skin able to discriminate a weight as low as a few grams. Moreover, the reconstruction algorithm has been initially tested to reconstruct four simultaneous pressure contacts and to prove the correct operation of the electronic skin when bent.

We attempted to go further the state–of-the-art of optical sensors using waveguide by adopting an information processing principle inspired by tomography, that coupled with the mechanical properties of the material chosen results in an unique e-skin, able to reconstruct the intensity profile of multiple contacts on its sensing area. We expect future implementations of this system to not be limited in the number of contacts. Moreover we show preliminary results of reconstruction of the shape of contact.

The active components are positioned around the periphery of the *sensing area*, and since they are not bearing the load, the skin is rugged and doesn't have drift of electrical characteristic with time. The power consumption is low because it scales with the number of components N and not with N^2^ as most artificial skins. This means that power consumption scales linearly instead of quadratically with the number of components, which is related to the desired spatial resolution.

Unlike previous approaches, that we define “point to point”, since an array of taxels is used to retrieve pressure information on a sensing area, our electronic skin can “sense” in every point of its surface as it adopts a distributed reconstruction method. This way the spatial resolution dramatically increases and it can be expected it to achieve values below 1 mm; the latter representing a good reference value for a human finger [33], the most sensitive touch sensor known.

All these characteristics open a plethora of specific applications of the electronic skin in robotic devices in general, including soft robots in which the gathering of information from a soft tactile skin is fundamental for their control. In parallel, the artificial skin can find ready application as a touch interface technology for consumer electronic devices, adding flexibility and true pressure detection to the functionalities available today.

Future works will include the development of a larger electronic skin, with a higher density of active components. Moreover, smaller components (SMD), will allow integration of a thinner elastomeric waveguide. As explained in the discussions section, such a device is expected to reach higher performances, especially in terms of spatial resolution and sensitivity. Further study will be dedicated to the theoretical analysis of the system, in order to account for different non-idealities in the real systems: for example, the mechanical deformations of the layer introduce non-trivial scattering effects, which need to be modeled in order to introduce opportune filters in the information processing phase, to further improve performances. Another issue that will be addressed is the spatial filtering of the elastic materials: knowing the modeling of this effect a reverse filter in the algorithm might be implemented, allowing resolving at the maximum spatial resolution the pressure contacts.
